# Evidence for partial melt in the crust beneath Mt. Paektu (Changbaishan), Democratic People’s Republic of Korea and China

**DOI:** 10.1126/sciadv.1501513

**Published:** 2016-04-15

**Authors:** Ri Kyong-Song, James O. S. Hammond, Ko Chol-Nam, Kim Hyok, Yun Yong-Gun, Pak Gil-Jong, Ri Chong-Song, Clive Oppenheimer, Kosima W. Liu, Kayla Iacovino, Ryu Kum-Ran

**Affiliations:** 1 Earthquake Administration, Pyongyang, Democratic People’s Republic of Korea.; 2 Department of Earth and Planetary Sciences, Birkbeck College, University of London, London WC1E 7HX, UK.; 3 Department of Geography, University of Cambridge, Cambridge CB2 3EN, UK.; 4 Environmental Education Media Project, Beijing 100025, China.; 5 U.S. Geological Survey, Menlo Park, CA 94025, USA.; 6 Pyongyang International Information Centre of New Technology and Economy, Pyongyang, Democratic People’s Republic of Korea.

**Keywords:** Volcano, Partial melt, receiver function, Seismology, crust

## Abstract

Mt. Paektu (also known as Changbaishan) is an enigmatic volcano on the border between the Democratic People’s Republic of Korea (DPRK) and China. Despite being responsible for one of the largest eruptions in history, comparatively little is known about its magmatic evolution, geochronology, or underlying structure. We present receiver function results from an unprecedented seismic deployment in the DPRK. These are the first estimates of the crustal structure on the DPRK side of the volcano and, indeed, for anywhere beneath the DPRK. The crust 60 km from the volcano has a thickness of 35 km and a bulk *V*_P_/*V*_S_ of 1.76, similar to that of the Sino-Korean craton. The *V*_P_/*V*_S_ ratio increases ~20 km from the volcano, rising to >1.87 directly beneath the volcano. This shows that a large region of the crust has been modified by magmatism associated with the volcanism. Such high values of *V*_P_/*V*_S_ suggest that partial melt is present in the crust beneath Mt. Paektu. This region of melt represents a potential source for magmas erupted in the last few thousand years and may be associated with an episode of volcanic unrest observed between 2002 and 2005.

## Teaser

Magma beneath Mt. Paektu may be associated with an episode of volcanic unrest that occurred between 2002 and 2005.

## INTRODUCTION

**Fig. 1:**
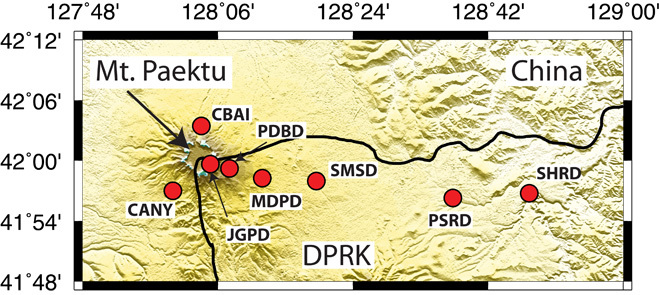
Station locations. Seismic stations used in the RF study (red circles). DPRK stations are Jang Gun Peak (JGPD), Paektu Bridge (PDBD), Mudu Peak (MDPD), Sin Mu Song (SMSD), Paek San Ri (PSRD), and Sing Hung Ri (SHRvD).

The Millennium Eruption of Mt. Paektu (known as Changbaishan in China), circa 946 AD (*1*), is one of the largest in the historical record (*2*) with an estimated 24 km (dense rock equivalent) of rhyolite and trachyte magma erupted (*3*). The eruption is thought to have formed the 5-km-wide caldera at its summit that hosts Lake Chon (also known as Tianchi) on the border with the Democratic People’s Republic of Korea (DPRK) and China (Fig. 1). Ground deformation and fluid geochemical anomalies drew attention to this enigmatic volcano from 2002–2005, a period of seismic unrest (*4*, *5*). This led to a unique collaboration between scientists from the DPRK, UK, and United States to understand the geological history and internal structure of the volcano (*6*). Here, we present receiver function (RF) estimates of the crustal structure beneath the volcano. The RF results suggest that significant amounts of melt are present in the crust beneath the volcano with a lateral extent of at least 20 km. These are the first images of the structure beneath the DPRK side of the volcano and the first characterization of the crustal structure anywhere beneath DPRK.

### Geological background

Mt. Paektu is composed of a trachybasalt shield (~2.8 to 1.5 million years ago), a trachyte stratocone (~1.0 to 0.04 million years ago), and a comendite ignimbrite (Holocene) sitting on top of Archean and Mesozoic granitic basement (*7*, *8*). The origins of the volcano remain enigmatic but seem to be related to the subduction of the Pacific plate below the Eurasian plate. Numerous seismic tomographic models show a stagnant slab within the transition zone beneath northeast China (*9*–*12*). These models, as well as results from joint RF/surface wave inversions (*13*), reveal the presence of low upper-mantle seismic velocities linked to the presence of hot, partially molten upper mantle beneath Mt. Paektu/Changbaishan. One interpretation of these low velocities is that they represent water released from the slab within the transition zone leading to a “big mantle wedge,” where upwelling occurs above the stagnant slab (*9*, *14*). More recently, tomographic images (*12*) and measurements of transition zone discontinuity depths (*15*) based on seismic data from northeast China have been interpreted as indicating a gap in the stagnant slab. The regional volcanism is explained as being caused by hot, sub-slab material rising through this gap into the upper mantle. Although there is no consensus view on the origins of volcanism at Mt. Paektu, the observations are all consistent with a source of partial melt in the mantle beneath the volcano.

Previous efforts to estimate the crustal structure beneath Mt. Paektu have been limited to the Chinese side of the volcano. Controlled-source seismology has shown the prevalence of low velocities in the lower crust beneath the volcano (*16*, *17*), but debate continues about the details of the structure. Controlled-source data suggest that the lowest velocities are present directly beneath the volcano (*16*), but other studies using the same data set suggest that lower velocities are located 30 to 60 km to the north (*17*). Magnetotelluric data and associated models reveal a region of high conductivity in the lower crust directly beneath the volcano (*18*) in a similar location to that of the low seismic velocities suggested by Zhang *et al.* (*16*). Additionally, forward-modeled gravity data indicate low densities in this region (*19*). Finally, RFs based on a few months of teleseismic observations recorded close to Mt. Paektu are consistent with a low-velocity lower crust (*20*). All these studies interpreted the anomalous lower crust as indicative of melt that may be linked to both the past eruptions and the recent unrest beneath the volcano.

### Recent unrest

The unrest from 2002 to 2005 was characterized by significant seismicity. Earthquakes were located independently by teams from DPRK (*21*) and China (*4*), and epicenters were shallower than 5 km. Ground deformation, measured by Global Positioning System and leveling on the Chinese side of the volcano, was another manifestation of the unrest. It was modeled by a Mogi source at 2 to 6 km depth (*4*). These observations were seen as indicative of recharge of a shallow magma chamber. Increases in He emissions at hot springs, with high ^3^He/^4^He (*R*/*R*_a_ of 5.6), were taken to suggest contribution from a deeper, possibly mantle, source (*4*, *5*). These results suggest the existence of a complex magma storage region with both shallow and deep crustal melt storage regions beneath Mt. Paektu.

## RESULTS

### Seismic data

We deployed six broadband seismometers in a linear profile from the caldera rim and extending 60 km to the east (Fig. 1). Station details are reported in Table 1. We focus here on the analysis of data collected in the first year of deployment (August 2013 to August 2014). We also include data from two stations deployed close to Mt. Paektu in China that have been previously used to generate RFs (*20*).

**Table 1:** Station details and *H*-κ stacking results.

**Station**	**Sensor**	**Latitude (°)**	**Longitude (°)**	**Elevation (km)**	***H* (km)**	***V*_P_/*V*_S_**	***V*_P_ (km s^−1^)**	**Number of RFs**
JGPD	ESP	41.994	128.083	2.648	—	—	6.2	23
PDBD*	40T	41.987	128.126	2.164	33±1 39±1	2.11±0.02 1.94±0.03	6.2	52
MDPD*	ESP	41.970	128.198	1.799	32±2 39±3	2.06±0.12 1.87±0.11	6.2	30
SMSD	ESP	41.966	128.318	1.443	36±1	1.84±0.03	6.5	38
PSRD	40T	41.938	128.622	1.101	35±1	1.76±0.05	6.5	6
SHRD	ESP	41.946	128.791	1.041	35±1	1.79±0.04	6.5	48

*These stations yielded two *H*-κ stacking solutions as shown in the columns for *H* and *V*_P_/*V*_S_. See the text for details.

### Receiver functions

**Fig. 2:**
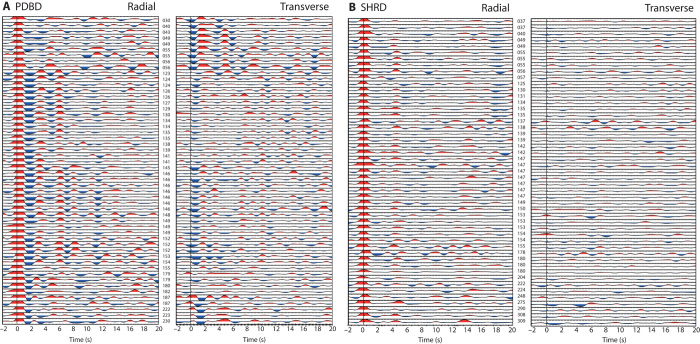
Example RFs. (**A** and **B**) All RFs generated at stations PDBD (A) and SHRD (B). Three-digit number between radial and transverse components refers to the earthquake back azimuth.

To estimate crustal structure, we used the RF technique, which attempts to isolate *P*-to-*s* conversions from major discontinuities in Earth by deconvolving the *P*-wave energy from the seismograms (*22*). Figure 2 shows examples from two stations, PDBD (close to the volcano) and SHRD (the easternmost station). A first-order observation is that the crustal structure far from the volcano appears much simpler than that directly beneath the volcano. SHRD, the easternmost station, shows a clear *Ps* arrival at ~4.5 s and clear reverberations at ~13 and 18 s. Also, these data show little transverse component energy for all back azimuths. RFs at station PDBD show much more structure. The clearest signal is a large negative peak at ~2 s with a number of coherent phases in the first 8 s. A great deal of energy is observed on the transverse component at these times as well, suggesting complex structure such as a dipping layer or anisotropy (*23*).

### *H-*κ stacking

**Fig. 3:**
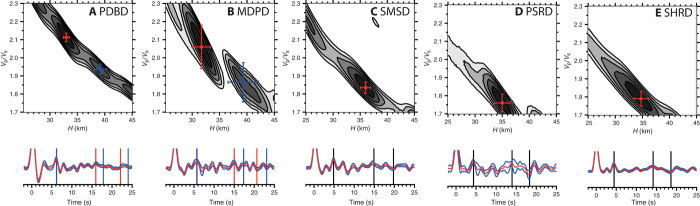
*H*-κ stacking results. (**A**) PDBD. (**B**) MDPD. (**C**) SMSD. (**D**) PSRD. (**E**) SHRD.

We estimated crustal thickness and the bulk crustal ratio of *P*-wave velocity to *S*-wave velocity (*V*_P_/*V*_S_) using the *H*-κ stacking technique (*24*). The three stations deployed far from Mt. Paektu (SMSD, PSRD, and SHRD) show simple *H*-κ stacking results (Fig. 3). Stations PSRD and SHRD are found to sit above a thick crust (35 ± 1 km) with a *V*_P_/*V*_S_ of 1.76 ± 0.05 and 1.79 ± 0.04, respectively (see Materials and Methods for details on how errors are quantified). Station SMSD shows a similar crustal thickness (36 ± 1 km) but with a slightly elevated *V*_P_/*V*_S_ (1.84 ± 0.03). The stations close to the volcano show more complicated *H*-κ stacking results (Fig. 3). Indeed, two solutions are suggested for MDPD and PDBD. Limiting the range of crustal thicknesses used in the grid search permits a more detailed investigation. We limit the range of crustal thickness search to 32 to 38 and 29 to 35 km for PDBD and MDPD, respectively, for the shallow result and 36 to 42 and 37 to 43 km, respectively, for the deeper result. We show that two consistent solutions are observed. One solution indicates a thinner crust with a very high *V*_P_/*V*_S_ (PDBD: 33 ± 1 km, 2.11 ± 0.02; MDPD: 32 ± 2 km, 2.06 ± 0.12) and a second solution indicates a thicker crust but with a lower *V*_P_/*V*_S_ (PDBD: 39 ± 1 km, 1.94 ± 0.03; MDPD: 39 ± 3 km, 1.87 ± 0.11). Unfortunately, the RF for JGPD was too complex (possibly because of local scattering effects) to yield a well-constrained solution. Also, the two stations in China (CBAI and CANY) provided too few data to produce good *H*-κ stacking solutions.

### RF migrations

**Fig. 4:**
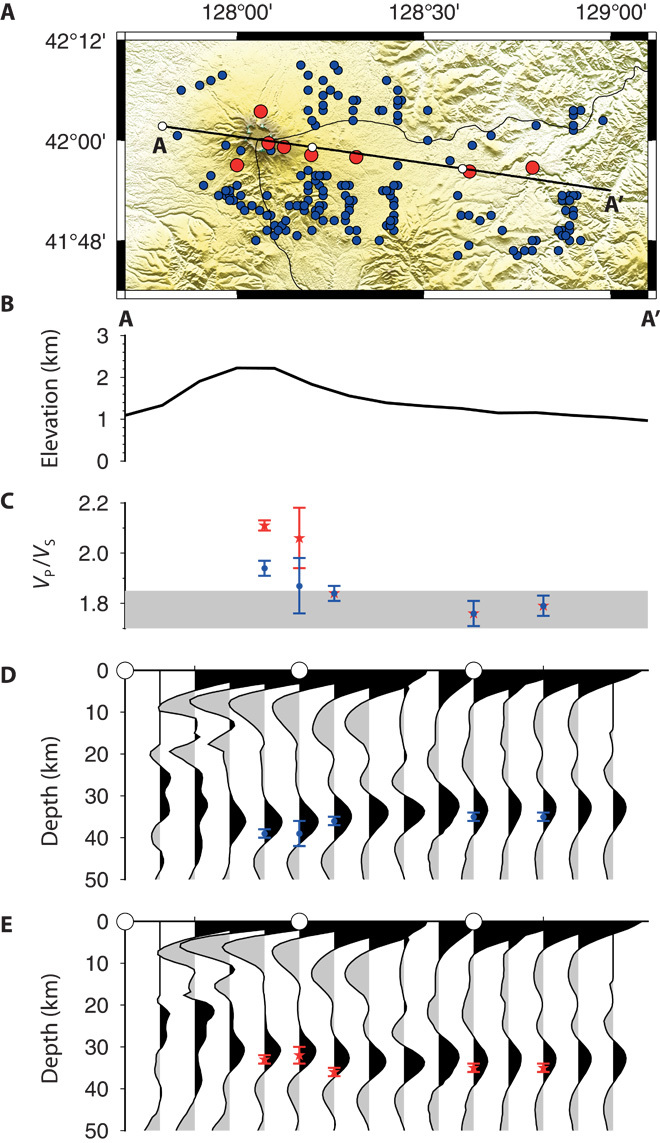
RF migrations beneath Mt. Paektu. (**A**) Map shows stations (red circles) and piercing points (blue circles) at the crust-mantle boundary (assuming a crustal thickness of 35 km and an IASP91 velocity model). (**B**) Elevation along profile A-A′ shown in (A). (**C**) *V*_P_/*V*_S_ estimated from *H*-κ stacking [blue circles are related to the crustal thickness solution in (D) and red stars are related to the crustal thickness in (E)]. (**D**) RF migrations assuming a crustal velocity with the lower *V*_P_/*V*_S_ solution at PDBD and MDPD. (**E**) RF migrations assuming a crustal velocity with the higher *V*_P_/*V*_S_ solution at PDBD and MDPD.

The final stage in our analysis was the migration of the RFs to depth based on our estimates of the crustal structure embedded within the one-dimensional IASP91 velocity model (*25*, *26*). Two migrations are shown in Fig. 4. These are based on the two solutions estimated in the *H*-κ stacking for stations close to the volcano. If the higher *V*_P_/*V*_S_ is assumed to be correct, then the crust must thin beneath the volcano. If a lower *V*_P_/*V*_S_ is assumed, then the crust thickens slightly beneath the volcano. A clear feature of both migrations is the strong negative peak seen at 5 to 10 km beneath the stations close to the volcano. In contrast, the crust farther away from the volcano is very simple.

## DISCUSSION

The stations far from the volcano suggest that the unperturbed crust in the northern DPRK is ~35 km thick with a *V*_P_/*V*_S_ of ~1.76 to 1.79. These are typical values of crustal thickness and *V*_P_/*V*_S_ for continental crust seen globally (*27*), and typical values found for the crust forming the Sino-Korean craton (*28*, *29*). However, the crust beneath the volcano is more complicated and has clearly been modified by the 3.5–million year history of volcanism in the region (*8*, *30*).

Two solutions are evident in the *H*-κ stacks, but the migrations emphasize that only one of these solutions can be correct. There is little evidence for multiple peaks in the data to suggest a complex lower crust, and the change in *V*_P_/*V*_S_ would suggest an extremely low *V*_P_/*V*_S_ in a 6- to 7-km-thick layer at the base of the crust. Previous studies of the crustal structure on the Chinese side of the volcano have suggested that the crust beneath Mt. Paektu is thicker than the surrounding region, reaching a thickness of ~40 km (*16*, *17*). This is similar to our deeper estimate, and thus is our preferred solution. Both these solutions indicate very high *V*_P_/*V*_S_ values (MDPD, 1.87; PDBD, 1.94). This finding is consistent with estimates of Poisson’s ratio from refraction studies that observed values as high as 0.3, equivalent to a *V*_P_/*V*_S_ of 1.88 (*31*) together with low seismic velocities (*16*, *17*) and high conductivities (*18*) in the lower crust. Experimental data indicate that typical *V*_P_/*V*_S_ values for crustal rocks range from 1.7 for silicic rocks to 1.87 for more mafic compositions (*32*) (Fig. 4). For Mt. Paektu, where the crust is likely to host abundant mafic rocks associated with magmatism, we would expect typical crustal *V*_P_/*V*_S_ values to lie between 1.76 (assuming our estimate of *V*_P_/*V*_S_ from PSRD represents the background continental crust composition) and 1.87. This suggests that compositional changes alone cannot explain our observations. Such high *V*_P_/*V*_S_ values exceeding 1.9 have been found for other volcanic regions [for example, Ethiopia (*26*, *33*, *34*)] where they have been interpreted as signifying the presence of partial melt. A recent study in Ethiopia has shown that the elevated *V*_P_/*V*_S_ is an artifact of anisotropy (*35*), and thus the high *V*_P_/*V*_S_ beneath volcanoes is likely due to the presence of aligned melt (for example, stacked sills or dikes within the crust). The high transverse component energy observed at stations close to Mt. Paektu (Fig. 2) lends some support to the interpretation of azimuthal anisotropy and thus the presence of vertically aligned melt. However, a more detailed study investigating back-azimuthal variations in *V*_P_/*V*_S_ is required to test this hypothesis. The *V*_P_/*V*_S_ ratio computed for station SMSD (1.84 ± 0.03), ~20 km from the volcano, remains elevated compared to stations assumed to lie above unaltered crust (1.76 to 1.79). This could reflect the presence of melt or a more mafic crust (*32*). Either way, partial melt is and/or was present at least 20 km from Mt. Paektu.

A second feature consistent with the presence of melt is the strong negative peak seen at ~2 s mapped to 5- to 10-km depth in the crust. This depth corresponds with that for (i) low velocities observed in the crust from refraction studies (*16*, *17*) and (ii) depths proposed for a magma source from modeling deformation observed during the episode of unrest (2 to 5 km) (*4*). This finding suggests that we may be seeing the top of a significant magma storage region in the shallow crust, but further tests, such as forward modeling to include the effects of anisotropy, are needed to provide further constraints.

The evidence for high *V*_P_/*V*_S_ throughout the crust close to the volcano combined with the coherent negative peak in RF at a depth comparable with previous estimates for depths of magma storage beneath Mt. Paektu suggests that partial melt is present beneath the volcano. The results show that a large region of the crust has been modified by magmatism associated with volcanism and that partial melt is likely to be present throughout a significant portion of the crust. This region may represent a potential source for magmas erupted in the historical period and possibly associated with the episode of unrest that occurred between 2002 and 2005.

## MATERIALS AND METHODS

### Seismic array

We used the data from six broadband seismic stations, provided by the Natural Environment Research Council (NERC) UK instrument pool SEIS–UK (*36*). Our seismic array consisted of four CMG-ESP (60-s natural period) and two CMG-40T (30-s natural period) Guralp seismometers. Seismometers were deployed in purpose-built vaults to reduce noise and to protect from the harsh winter weather conditions. All data were sampled at 50 Hz.

### RF analysis

We generated RFs using the extended time multitaper technique (*37*). We manually picked the *P*-wave arrival and used an ~80-s window to perform deconvolution. We applied a frequency domain low-pass cosine taper with a 1.0-Hz cutoff frequency to band-limit the data. We used the recordings of teleseismic earthquakes (>5.5 mb) from distances between 30° and 90°. In general, signal quality was very good; 19% of earthquakes analyzed produced good quality RFs (assessed through a visual check, where a sharp peak on the vertical component coupled with coherent energy on the radial and transverse component). This resulted in a total of 197 RFs.

### *H*-κ stacking

The arrival times of a *Ps* conversion and reverberations (*PsPs*, *PpSs*, and *PsPs*) from a discontinuity such as the Moho depend on the incoming slowness, the *P*-wave velocity in the crust, the crustal thickness (*H*), and the ratio of the *P*-wave velocity to the *S*-wave velocity (κ). The slowness was calculated from the earthquake location. We could estimate the average *P*-wave velocity from nearby controlled-source experiments (*16*, *17*) (Table 1), which means it was possible to grid-search over values of *H* and κ, summing the amplitudes at the predicted times to estimate the crustal thickness and bulk crustal *V*_P_/*V*_S_ (*24*). We used an *H* range of 25 to 45 km in intervals of 0.25 km and a κ range of 1.7 to 2.3 in intervals of 0.0075. We weighted all three phases in the stack equally. This technique assumes horizontal layering of the crust and isotropy, both of which can affect the accuracy of the *H*-κ stacking technique (*33*, *35*). To estimate the errors, we incorporated a bootstrap technique (*38*) that randomly selects RF data and estimates *H* and κ. We repeated this 2000 times to test the stability of the result. We also tested the error in our assumption of the average *V*_P_ by changing the value by 0.1 km s^−1^. Whichever error was the largest was used as the error in our solution.

### Common conversion point migration

To investigate the lateral variations in the data, we performed a common conversion point migration of the data. We used the method of Angus *et al.* (*25*) with slight modifications (*26*) to incorporate the *H*-κ stacking constraints of this study. We back-projected the RF energy along the IASP91 ray path, but changed the crustal thickness and the average *P*- and *S*-wave velocity based on constraints from nearby refraction experiments (*16*, *17*) and the estimates of *H* and κ from this study. This means that each station had an individual velocity model. All migrations were shown with respect to sea level. The model was parameterized into 10-km bins, and the radius was defined by the Fresnel zone (and thus increased with depth).
